# The Use of an Electronic Health Record Patient Portal to Access Diagnostic Test Results by Emergency Patients at an Academic Medical Center: Retrospective Study

**DOI:** 10.2196/13791

**Published:** 2019-06-28

**Authors:** Brody Foster, Matthew David Krasowski

**Affiliations:** 1 Department of Pathology University of Iowa Hospitals and Clinics Iowa City, IA United States

**Keywords:** consumer health informatics, diagnostic imaging, electronic health records, medical informatics, minority groups, patient portals, proxy, user-computer interface

## Abstract

**Background:**

Electronic health record (EHR) patient portals provide a means by which patients can access their health information, including diagnostic test results. Little is known about portal usage by emergency department (ED) patients.

**Objective:**

The study aimed to assess patient portal utilization by ED patients at an academic medical center using account activation rates along with the rates of access of diagnostic test results (laboratory results and radiology reports), analyzing the impact of age, gender, and self-reported patient race.

**Methods:**

This institutional review board–approved retrospective study was performed at a 60,000-visits-per-year university-based ED. We utilized EHR data reporting tools to examine EHR portal activation and utilization for all patients who had at least one ED encounter with one or more diagnostic tests performed between October 1, 2016, and October 1, 2017. The total dataset for laboratory testing included 208,635 laboratory tests on 25,361 unique patients, of which 9482 (37.39%) had active portal accounts. The total dataset for radiologic imaging included 23,504 radiology studies on 14,455 unique patients, of which 5439 (37.63%) had an active portal account.

**Results:**

Overall, 8.90% (18,573/208,635) of laboratory tests and 8.97% (2019/22,504) of radiology reports ordered in the ED were viewed in the patient portal. The highest rates of viewing of laboratory and radiology results were seen for those who were female, were aged 0 to 11 years (parent or guardian viewing by proxy) and 18 to 60 years, and self-reported their race as Caucasian or Asian. The lowest rates were for those who were teenagers, aged older than 81 years, African American/black, and Hispanic/Latino. Infectious disease, urinalysis, and pregnancy testing constituted the highest number of laboratory tests viewed. Magnetic resonance imaging reports were viewed at higher rates than computed tomography or x-ray studies (*P*<.001). Approximately half of all the diagnostic test results accessed by patients were reviewed within 72 hours of availability in the patient portal (laboratory results: 9904/18,573, 53.32% and radiology reports: 971/2019, 48.1%). On the other extreme, 19.9% (3701/18,573) of laboratory results and 31.6% (639/2019) of radiology reports were viewed more than 2 weeks after availability in the portal.

**Conclusions:**

The data highlight the relatively low use of a patient portal by ED patients and existing disparities between patient groups. There can be wide lag time (months) between result/report availability and access by patients. Opportunities for improvement exist for both activation and more robust utilization of patient portals by ED patients.

## Introduction

### Context

Electronic health records (EHRs) are common among many industrialized countries and provide a way for patient health information to be stored and accessed in an efficient and secure fashion [1-4]. EHRs can also provide a tool for patients to access their own health information. Web-based EHR portals (also known as *patient portals*) have become increasingly popular over the past decade [5-11] and have been promoted by legislative and regulatory initiatives such as the Health Information Technology for Economic and Clinical Health (HITECH) act in the United States [12-14]. HITECH aims to improve the overall quality of health care in the United States by encouraging patient involvement in medical decisions. HITECH has also included financial incentives and penalties that have led to EHR implementation in over 86% of US physician practices [1]. Health care institutions have found EHR patient portals to be a popular feature for patients [5,9,10,15-17]. Patient portals can theoretically reduce time-consuming tasks for the health care team such as phone calls (especially if patients are not readily accessible) and mailing hard copies of letters.

### Background

Previous studies have shown that patients have a positive perception of using patient portals to become more fully engaged in their own medical decisions [5,10,15-18]. Patient portal use has also been linked to improvements in health care coordination, decreased costs, and better communication between patients and health care providers [19-22]. Appointment scheduling, medication refills, pathology results, radiology reports, and direct messaging with the health care team are some of the most popularly accessed features in patient portals [5,8,9,16,23-25]. Multiple studies in the United States and Europe have shown that the highest users of patient portals tend to be Caucasian, female, and adults aged 25 to 50 years [8,9,16,23,26-30]. However, there are factors that can impact acceptance and use of patient portals. These include language fluency, health care literacy, comfort and familiarity with using the internet, access to broadband internet access, support and assistance from family and friends, and encouragement by the health care team [6,30-35]. The published literature on patient portals has mostly focused on the use of these portals in the outpatient setting, where the full functionality of the portals can be utilized (eg, messaging with providers, prescription refills, scheduling appointments, and viewing provider reports and diagnostic test results). In addition to outpatient applications, some recent literature highlights the use of patient portals in the inpatient setting (eg, sending questions to the health care team and ordering hospital meals) [34,36-39].

In contrast, there have been few studies of patient portal usage by emergency department (ED) patients [40,41]. A previous survey indicated ED physicians’ concerns with the release of diagnostic test results to patients while acknowledging some benefits [36]. The population of patients seen in the ED presents some challenges for promoting patient portal usage as these patients may not seek regular medical care at the institution or health network affiliated with the ED. Popular patient portal features such as messaging with the clinical team, pharmacy refills, and appointment scheduling may be of little or no benefit to ED patients. On the contrary, the use of a patient portal affords patients easy access to diagnostic test results and empowers patients to transfer information to other health care facilities. Perhaps, most importantly, portals can allow ED patients to review diagnostic test results and other EHR information (eg, discharge summaries) in their own time; this may be especially advantageous for ED visits for medical issues that can impact cognition and understanding during the acute event.

We have previously analyzed general patterns of patient access of diagnostic test results through the patient portal (Epic MyChart) used at our institution, a Midwestern academic medical center (University of Iowa Hospitals and Clinics; UIHC) [41,42]. One previous study analyzed broad patterns of diagnostic test release and patient access, in particular, focusing on the mechanism and timing of results transmittal to the patient portal [41]. This previous study provides details on the release categories for diagnostic tests and also describes the challenges and potential safety issues encountered at UIHC with patient access of their own test results/reports. A later study focused in detail on outpatient use of the patient portal and identified disparities in portal activation and usage by underrepresented minorities [42].

### Objectives

In this study, we analyzed the activation and use of a patient portal by patients at our 60,000-visits-per-year, university-based ED that serves as a regional level 1 trauma center. We focused on patient viewing of laboratory results and radiology reports (collectively referred to as *diagnostic tests*) ordered within the ED, as these are patient portal functions frequently used by ED patients at our institution. For viewing of the diagnostic test results, an analysis was performed for both the total cohort (including those who have never activated a patient portal account) and the more limited subset with active patient portal accounts. Analysis of the 2 populations helps address the separate impact of 2 broad barriers in patient portal usage: getting patients to activate accounts and, once activated, to utilize portal functionality such as viewing diagnostic test results.

Some popular functions of the patient portal in the UIHC outpatient setting are not applicable to the ED patient population. In particular, scheduling of ED visits is not available in the UIHC portal. Prescription refills via the patient portal are also not applicable as ED prescriptions at UIHC are almost always written without refills. In addition, messaging of the health care team via the portal, although possible for ED patients, has not been offered as a preferred means of ED follow-up communications compared with telephone calls. In the period of retrospective analysis, ED patient messaging for follow-up questions was of negligible volume (<15/month). Our hypothesis was that utilization of the patient portal for diagnostic test result viewing in the ED would be lower than previously published rates in our outpatient population but would show similar variation based on age, gender, and self-reported race.

## Methods

### Study Setting

This study was a retrospective analysis of EHR patient portal records at the UIHC ED between October 1, 2016, and October 1, 2017. The institution has 811 inpatient beds at the main campus and outpatient clinics throughout the local geographic region. UIHC is a tertiary/quaternary care facility that serves as a regional center for specialty care.

### Patient Portal

The institution uses Epic (Epic, Inc) as the EHR and adopted its associated patient portal (MyChart) in 2010 [41]. To set up a patient portal account, patients receive information in their after-visit summary documents that include a MyChart activation code and instructions for activating and using their portal account. Patients may also request an activation code online. Parents and legal guardians can activate and manage portal accounts of children aged 11 years and younger by proxy with full functionality. Proxy functionality is limited for children aged 12 to 17 years (eg, parents and guardians cannot access diagnostic test results) and ends when children turn 18 years. With documentation, legal guardians of adult dependent patients may obtain proxy access.

The MyChart graphical user interface has evolved over time, but here, we summarize some of the basic elements of the one in use at UIHC during the period of retrospective analysis. The login screen identifies as MyChart for University of Iowa Health Care and allows for login using username and password, a link to sign up for access, or option for username or password recovery. Once logged in, the user has menu options for *Health* (eg, test results, health summary, current health issues, medications, allergies, and immunizations), *Visits* (viewing past, current, or future appointments and scheduling or canceling appointments), *Messaging* (inbox, ability to send and receive messages from health care team, and request for prescription refills), *Billing*, *Resources* (eg, medical library), and [User] *Profile*. Parents or legal guardians will see an option for accessing MyChart for their dependents. The Profile menu allows for various options for receiving notifications when new information (eg, diagnostic test results, medical documents, prescriptions, and bills) is available. Diagnostic test results can be accessed directly off the *Health* menu. For any given laboratory test, there is the ability to view past results (grouped into columns by date) and also graph/trend past results.

The institutional patient portal allows patient access to laboratory test results and radiologic imaging reports [41,42]. Authorized health care providers can manually *release* diagnostic test results to the patient portal once the results are finalized in the EHR; the results then immediately appear in the patient portal. Results that do not get manually released will *autorelease* based on a defined schedule. As mentioned above, patients can set up an email or other method for notifications when diagnostic test results become available in MyChart.

Common laboratory tests (eg, basic electrolyte and renal/kidney tests, cardiac troponin, complete blood count, coagulation tests, and urinalysis) autorelease following a 1–business day delay [41]. Radiologic imaging results and more sensitive laboratory tests (eg, sexually transmitted disease testing and genetic studies) autorelease with a 4–business day delay. A small number of other tests (eg, HIV testing and Huntington disease genetic studies) never release to the patient portal (*do not release* status). Other than HIV testing, diagnostic tests that do not release to the patient portal are very rarely ordered in the ED (the no-release status for HIV results in the patient portal allows for compliance with the State of Iowa law that requires patient counseling for reporting of positive HIV results) [41].

### Data Retrieval and Analysis

There was a years’ lag time between the retrospective period of diagnostic test ordering and our subsequent data analysis, allowing for longer-term assessment of patient portal access that may have occurred months after the ED patient encounter. This study was approved by the local institutional review board with a waiver for informed consent and was also part of a broader quality improvement initiative to improve patient use of the UIHC patient portal.

The study involved 2 primary measures. The first primary outcome involved analyzing patient portal activation rates for patients seen at the medical center ED who had one or more diagnostic tests performed. *Active* portal status was defined as those patients (or proxy) that registered their EHR portal account online with an activation code provided by the medical center. *Inactive* portal status was defined as those who had not used an activation or who did not elect to receive one. The second measure involved analysis of viewing patterns of diagnostic test results within the patient portal. Laboratory tests were divided into the categories of chemistry (including blood gas analysis), hematology (including blood count and coagulation testing), and microbiology (including blood and urine cultures along with more targeted infectious disease testing). Radiologic imaging was divided into the broader categories of computed tomography (CT), magnetic resonance imaging (MRI), and x-rays. Patient portal active status and viewing of diagnostic test results did not distinguish between patient or proxy access to the portal.

Epic Reporting Workbench (RWB) is a reporting tool within the EHR that can retrieve data based on specified query parameters [43]. The analysis for this study used the *Search Orders* template within RWB to look for laboratory test and radiologic imaging orders covering dates from October 1, 2016, to October 1, 2017, that were ordered during ED encounters and then completed (ie, excluded orders cancelled before being completed). The base functionality of the RWB search retrieves each specific order, patient identifier, and date/time of order. The search was enhanced to capture additional data fields associated with each order: patient gender, age, self-declared patient race, patient portal activity status (inactive or activated), when the diagnostic test result released to MyChart, whether the specific diagnostic test results were viewed in MyChart, and, if viewed, the date/time of MyChart viewing. Owing to the sheer number of diagnostic tests, the orderable menus for laboratory tests and radiologic imaging available during the retrospective time period were broken up into 20 separate search templates that were searched 2 weeks at a time to cover the year of retrospective analysis. The outputs of the searches were downloaded to a spreadsheet format, with each row containing a unique test order and the additional data fields described above. The laboratory test and radiologic imaging data were separately combined, with subcategories assigned to specific diagnostic tests (eg, x-ray or magnetic resonance imaging). Spreadsheet analysis included pivot table analysis and was able to determine endpoints such as rates of portal activation and viewing rates of specific diagnostic tests associated with patient demographic features. The date/time stamps associated with the result release from the EHR to MyChart and patient access of results allowed for calculation of lag time between result release and viewing.

The categories listed for self-declared race were based on their primary response documented in the EHR. The groups included African American/black, Asian, Hispanic/Latino, other, and white. The category listed as *other* was for all patients that did not fall into any of the others. This included patients that indicated Native American, Alaskan, declined to answer, multiracial, Pacific Islander, Hawaiian, or unknown.

### Statistical Analysis

The dataset for laboratory tests included 208,635 tests performed in the ED on 25,361 unique ED patients, of which 9482 (37.39%) had an active portal account. Note that the laboratory test dataset is limited only to those patients who had one or more laboratory tests performed in the ED. Analysis was performed separately for the total cohort (includes those who have not activated their patient portal account and thus could not view results) and for the subset limited only to those who had an active patient portal account. Analysis of the 2 populations helps address 2 variables that influence viewing of laboratory tests: (1) activation of the portal and (2) use of the portal once an account is activated. We conducted chi-square tests to compare view rates (viewed or not viewed) of laboratory tests by age category, gender, and self-declared race. As a total of 42 comparisons were made, the Bonferroni correction was used, and the significance level was set at .0011.

A similar approach was used for the radiologic imaging data. The cohort that had radiologic imaging studies performed in the ED included 23,504 studies on 14,455 unique patients, with 37.61% (5436) of these patients having an active patient portal. This imaging data only included patients who had one or more imaging studies ordered while in the ED. We conducted chi-square tests to compare view rates (viewed or not viewed) of imaging reports by age category, gender, and self-declared race. The Bonferroni correction was used, and the significance level was set at .0011.

## Results

### Rates of Patient Portal Activation and Viewing of Laboratory Tests by Patients Seen in the Emergency Department

During the retrospective analysis period, 208,635 tests on 25,361 unique patients were performed in the ED, of which 37.39% (n=9482) of these patients had an active patient portal account. In terms of the number of ED visits, 80.56% (n=20,430) unique patients had a single ED visit with laboratory testing, 16.04% (n=4069) had 2 or 3 ED visits, 3.16% (n=802) had 4 to 10 ED visits, and only 0.24% (n=60) had more than 10 ED visits. Activation rates were lower for those with only a single ED visit (7312/20,430, 35.79%) compared with either those with 2 to 3 ED visits (1770/4069, 43.50%; *P*<.001) or 4 or more ED visits (368/862, 42.7%; *P*<.001). Females (5546/13,149, 42.18%) were significantly more likely to activate their portal than males (3897/12,212, 31.91%; *P*<.001).

Activation rates were highest for those with self-declared race as Asian (262/451, 58.1%) and white (8155/20,637, 39.52%) and lower for African American/black (491/2254, 21.78%; *P*<.001 compared with white), Hispanic/Latino (333/1257, 26.49%; *P*<.001 compared with white), and other (241/762, 31.6%; *P*<.001 compared with white). Activation rates for patients aged 18 to 70 years were 41.61% (7593/18,246); all subgroups within that broad age range (18-25 years, 26-30 years, 31-40 years, 41-50 years, 51-60 years, and 61-70 years) were at least 39.1%. Activation rates were lower for those aged 0 to 11 years (437/1626, 26.88%), 12 to 17 years (215/1129, 19.04%), 71 to 80 years (785/2447, 32.08%), 81 to 90 years (385/1568, 24.55%), and older than 91 years (67/345, 19.4%; *P*<.001 for each subgroup compared with 18-70 years).

**Figure 1:**
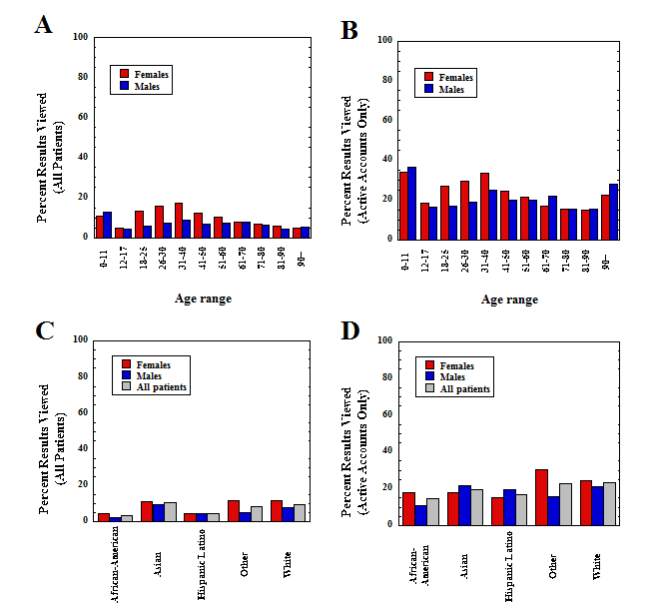
View rates of emergency department laboratory tests and variation by age, gender (a and b), and self-declared race (c and d). Population includes all patients who had at least one laboratory test performed during period of retrospective analysis. Data based on 25,361 unique patients (2254 African American/black, 451 Asian, 1257 Hispanic/Latino, 762 other, and 20,637 white; 13,157 female and 12,204 male) and 208,635 tests (103,548 for female patients and 105,087 for male patients). Bars show the percentage of results viewed within each subcategory.

Figure 1 shows the rate of laboratory test viewing in the patient portal, broken down by age and gender, for all patients and limited to only those with an activated patient portal account. Overall, 8.91% of laboratory tests (18,573/208,635) ordered in the ED were viewed in the patient portal. Several broad trends were evident. In general, females viewed laboratory test results at higher rates (10.73%, n=11,049) than males (7.20%, n=7524; *P*<.001), with viewing rates by females approximately double or more than that of males in the 18 to 50 years age range, the years of peak viewing. Females viewed laboratory results at rates higher than males for every age subgroup between 18 and 90 years with the exception of 71 to 80 years (*P*<.001 for each comparison; *P*=.10 for 71 to 80 years). Viewing rates were similar between males and females in other age ranges and lowest overall in the 12 to 17 years category. For the patients that had active portal accounts, females viewed at rates significantly greater than males overall (*P*<.001) and for all age categories between 18 and 50 years (*P*<.001 for each subgroup in this age range).

Figure 1 also breaks down the laboratory viewing data by gender and self-reported race, separating into all patients and limited to those with an active portal account. One notable trend is that Hispanic/Latino and African American/black groups had the lowest viewing rates compared with the other race categories, which is especially evident in the overall patient graph (*P*<.001 for each, compared with white). Both white and Asian viewing rates approximate the overall rates for the ED population, reflecting that these 2 categories constitute a majority of the population in the ED. The differences between the race categories are less striking when restricted to not only those with active portal accounts but also those with significantly lower viewing rates for Hispanic/Latino and African American/black groups (*P*<.001 for each, compared with white). African American/black males had the lowest viewing rates of any subgroup.

Multimedia Appendix 1 shows a breakdown of data by self-reported race and age. For the total population with laboratory testing ordered in the ED (25,361 unique patients), African American/black and Hispanic/Latino groups had lower laboratory test viewing rates in every age category except 26 to 30 years for Hispanic/Latino (*P*<.001 for each subgroup compared with a comparable white subgroup). Differences were less pronounced when examining only the subset with activated portal accounts (9482 unique patients). Nevertheless, African African/black individuals had lower view rates for all categories compared with white (*P*<.001). Hispanic/Latino individuals were lower than white in all age categories (*P*<.001) except 0 to 11 years and 18 to 25 years.

Table 1 shows the laboratory tests with the highest frequency of viewing that were ordered in the ED. Infectious disease testing [*Chlamydia trachomatis* polymerase chain reaction (PCR), *Neisseria gonorrhoeae* PCR, blood culture, and urine culture] and serum human chorionic gonadotropin (generally ordered for pregnancy testing) accounted for 5 of 10 most highly viewed tests. In contrast, examples of highly ordered tests with very low view rates (<5.0% overall) include arterial blood gas, chloride, and plasma ethanol. The broad categories of chemistry, hematology, and microbiology tests had similar view rates (Table 1).

**Table 1:** Viewing rates of laboratory tests by emergency department patients.

Laboratory category or test			Laboratory tests viewed		
			N		n (%)
**Broad category**					
	Chemistry	120,047		10,499 (8.75)	
	Hematology	64,244		5804 (9.03)	
	Microbiology	24,330		2270 (9.33)	
**Specific laboratory test (at least 100 results for active portal users)**					
	Urine culture	729		106 (14.5)	
	D-dimer	1273		178 (13.98)	
	*Chlamydia trachomatis* polymerase reaction (PCR)	940		127 (13.5)	
	Blood culture	4250		570 (13.41)	
	Human chorionic gonadotropin, serum	665		88 (13.2)	
	*Neisseria gonorrhoeae* PCR	943		121 (12.8)	
	C-reactive protein	4679		542 (11.61)	
	Total protein	348		39 (11.2)	
	Complete blood count with differential	26,493		2913 (11.00)	
	Gamma-glutamyl transferase	432		47 (10.9)	
	Urinalysis	2976		311 (10.45)	
	Basic metabolic panel (“Chem 8”)	23,536		2414 (10.26)	
	Amylase	6052		614 (10.14)	
	Thyroid-stimulating hormone with reflex	4474		422 (9.43)	
	Bilirubin, total	884		78 (8.8)	
	Alkaline phosphatase	866		74 (8.5)	
	Hemoglobin, glycosylated (A_1c_)	659		56 (8.5)	
	Creatinine, plasma	567		47 (8.3)	
	Aspartate aminotransferase	1350		110 (8.14)	
	Drug of abuse screen—urine panel	4343		345 (7.94)	

### Rates of Patient Activation and Viewing of Radiologic Imaging Reports by Patients Who Had Imaging Performed While in the Emergency Department

**Figure 2:**
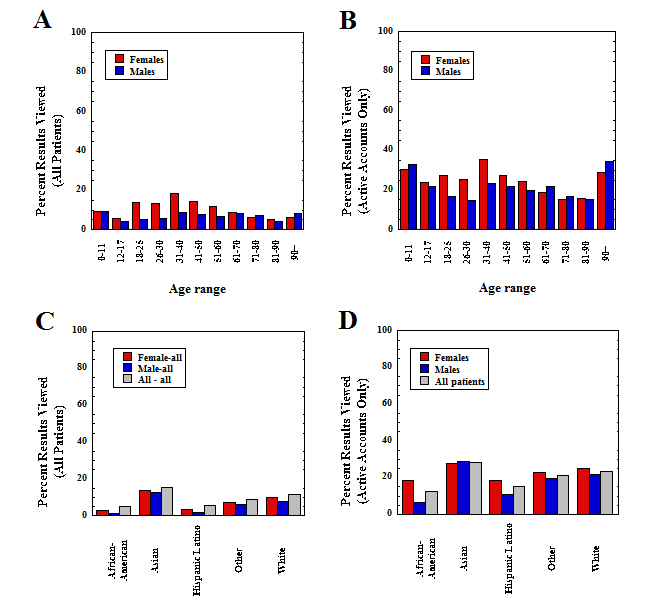
View rates of emergency department radiology tests and variation by age, gender (a and b), and self-declared race (c and d). Population includes all patients who had at least one radiologic imaging study performed during period of retrospective analysis. Data based on 14,455 unique patients (1274 African American/black, 220 Asian, 666 Hispanic/Latino, 407 other, and 11,888 white; 7079 female and 7376 male) and 23,504 radiology studies (11,202 for female patients and 12,302 for male patients). Bars show percent of results viewed within each subcategory.

During the retrospective analysis period, the cohort that had radiologic imaging studies performed in the ED included 23,504 studies on 14,455 unique patients, with 37.61% (5436/14,455) of these patients having an active patient portal account. Figure 2 shows the rate of radiologic imaging report viewing in the patient portal, broken down by age and gender for all patients and limited to only those with an activated patient portal account. Overall, 8.97% (2019/22,504) of the reports from radiologic imaging studies ordered in the ED were viewed in the patient portal.

The overall pattern of viewing with respect to age and gender showed similar trends to those described above for laboratory testing. Highest rates of viewing were seen in the age ranges of 0 to 11 years, 18 to 50 years, and older than 90 years. Females viewed imaging reports at rates higher than males both overall (*P*<.001) and for every age subgroup (overall cohort) between 18 and 60 years (*P*<.01 for each comparison). For those with active portal accounts only, females viewed imaging reports at higher rates than males in all age categories between 18 and 60 years (*P*<.001 for all groups except *P*=.004 for 26-30 years, *P*=.03 for 41-50 years, and *P*=.03 for 51-60 years).

Figure 2 also breaks down the radiology report viewing data by gender and race, separating into all patients and limited to those with an active portal account. Trends were very similar to the laboratory result viewing data (Figure 1), with the lowest viewing rates in the Hispanic/Latino and African American/black groups, especially in the overall patient graph (*P*<.001 for each compared with white). Similar to laboratory results, the differences between the race categories are less striking when restricted to not only those with active portal accounts but also with significantly lower viewing rates for Hispanic/Latino and African American/black groups (*P*<.001 for African American/black compared with white and *P*=.003 for Hispanic/Latino compared with white). Also similar to laboratory results, African American/black males had the lowest viewing rates of any subgroup.

Overall, MRI studies were reviewed at higher rates (329/2796, 11.77%) compared with CT scans (859/8957, 9.59%) and x-rays (911/11,750, 7.75%; *P*<.001 for MRI compared with either CT scan or x-ray for all ED patients). Table 2 shows the most viewed radiology studies with highest rates of viewing that were ordered in the ED, limited to studies with at least 50 orders in the retrospective analysis period. MRI studies (brain and spine imaging) and CT scans (abdomen/pelvis, chest, and head/neck) constituted 8 of the 10 tests in the patient portal with the highest frequency of viewing, with some studies viewed by over 30% (eg, MRI cervical spine with contrast: 25/73, 34%) of those with active portal accounts.

**Table 2:** Viewing rates of radiologic imaging reports by emergency department patients.

Radiologic imaging category or test		Imaging reports viewed	
		N	n (%)
**Broad category**			
	Magnetic resonance imaging (MRI)	2796	329 (11.77)
	Computed tomography (CT) scan	8957	859 (9.59)
	X-ray study	11,750	911 (7.75)
**Specific radiologic imaging study (at least 50 studies for all patients)**			
	MRI cervical spine with contrast	136	27 (19.9)
	CT abdomen and pelvis without contrast	837	131 (15.7)
	MRI brain—axial T2	188	27 (14.4)
	CT abdomen and pelvis with contrast	2106	299 (14.19)
	CT chest with contrast	175	22 (12.6)
	MRI brain with contrast	1774	215 (12.12)
	X-ray chest—anteroposterior and lateral	6418	644 (10.03)
	MRI cervical spine without contrast	240	21 (8.8)
	X-ray C-spine—anteroposterior and lateral	186	15 (8.1)
	MRI brain without contrast	274	21 (7.7)
	X-Ray abdomen— anteroposterior supine	468	35 (7.5)
	CT brain without contrast	5137	360 (7.01)
	X-ray chest anteroposterior/posteroanterior	3441	164 (4.77)
	X-ray pelvis—anteroposterior	1109	46 (4.14)

### Time to Access Results in Patient Portal

**Figure 3:**
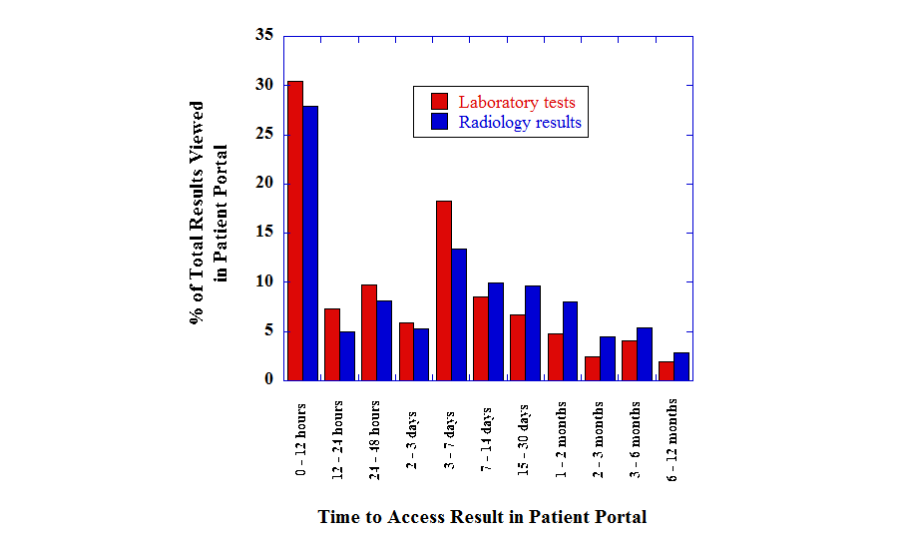
Time in which patient (or proxy) accesses laboratory test or radiologic imaging results after release to the patient portal. The data are divided into various time categories, summarizing the percentage of total results viewed in those categories.

Figure 3 shows how quickly patients accessed laboratory results or radiology reports after release into the patient portal. Only data from results that were accessed in the patient portal are included in Figure 3. There was a wide variability in how fast the results were accessed: laboratory results (mean 17.1, SD 42.7 days; median 2.3 days; minimum 0.9 min, maximum 359 days) and radiology reports (mean 24.0, SD 48.3 days; median 3.7 days; minimum 0.9 min, maximum 336 days). Compared with radiology results, laboratory tests tended to be more frequently viewed in time categories less than 1 week (*P*<.001). Nearly half of all results accessed in the patient portal are reviewed within 72 hours of release to the patient portal (laboratory results: 9904/18,573, 53.32%; radiology reports: 971/2019, 48.1%). On the other extreme, laboratory results and radiology reports viewed after 2 weeks constituted 19.93% (3701/18,573) and 971/2019, 48.1%, respectively, of the total views.

## Discussion

### Principal Findings

In this study, we show that activation and utilization of an EHR portal for viewing of diagnostic test results by patients at an academic medical center ED was the highest for those who were female, who were aged 0 to 11 years and 18 to 60 years, and with self-reported race as Caucasian or Asian. The lowest rates were for those who were teenagers, aged older than 81 years, African American/black, and Hispanic/Latino.

Patient portals are an increasingly popular feature for institutions using EHRs, offering a potentially more convenient way for patients to access their health information, coordinate appointments, and communicate with their health care team [5-11]. Previous studies have shown that patients who actively use portals are more likely to be female, be young to middle-aged adults, be English speaking, be Caucasian, have better health care literacy, and be in better health than nonusers [8,9,16,23,26-30,44]. Other studies have shown disparities in the use of EHR portals by underrepresented minorities [45-47]. There are complex individual factors that influence patient portal adoption [48]. The match between the design of the portal and patient expectations influences adoption and continued use [49], and some institutions have extensively incorporated stakeholder input into the portal design [50]. An example of incorporating user input to improve the adoption and use of MyChart is a project at the Johns Hopkins School of Medicine that trained volunteers to educate adolescent and young adult patients about the patient portal and to then facilitate enrollment and, for those interested, downloads of the MyChart mobile app [51].

In this study, we focused on the use of the patient portal for accessing diagnostic test results in an ED setting, comparing overall view rates (impacted by portal *nonusers* in addition to those with active accounts) with view rates among active portal users. The trends between laboratory results and radiology reports are very similar and will be discussed together. The highest use for both types of results occurred in the following groups: females; ages 0 to 11 years and 26 to 60 years; and white, Asian, and other self-declared race categories. Disparities between African American/black and Hispanic/Latino groups were more pronounced when viewing the overall patient population compared with just the subgroup that has an active portal account, demonstrating that low activation rates are a significant factor driving overall low viewing rates for African American/black and Hispanic/Latino patients in our study.

There are multiple approaches to reducing disparities in patient portal adoption. One positive step has been the development of multilingual patient portals, as described for a California health system serving a predominantly Spanish-speaking population [52]. Other factors that may influence portal usage include lack of broadband internet access, ownership of smartphones, and presence of family members or friends to assist with portal usage [33,45-47]. Initiatives to promote portal activation and usage should take into account these potential barriers. Future studies can also focus on the association between particular diseases/diagnoses and the use of the patient portal in the ED and other settings.

The rates of patient portal viewing of diagnostic tests in the ED in this study are considerably lower than in our previous study, which focused on outpatient clinics [42]. For outpatients, portal account activation rates at UIHC were 39.9% for females and 31.9% for males; the overall viewing of outpatient diagnostic tests results was nearly 40%, with some subgroups exceeding 55%. Very little has been published about how ED patients use portals, and our institution is probably similar to many others in having the broadest functionality of the patient portal for the ambulatory care setting. Efforts to promote patient portals in the ED can focus on the benefits of accessing information such as diagnostic testing and discharge summaries. This may help patients better understand their conditions as well as bring information and questions to future health care encounters. A visit in the ED may also be someone’s first exposure to a patient portal that can be used in other health care settings.

There has been increasing research related to patient portal usage for the pediatric population [53,54]. In this study, viewing of diagnostic test results in the 0 to 11 years patient age range (when parents/guardians have unrestricted proxy access) was similar to that in the 18 to 60 years range, with the exception that the access rates did not vary based on the gender of the child. The dataset did not capture which parent or guardian accessed the child’s results, so it is unknown if there are gender differences in proxy access or how often 2 parents/guardians view results together. Age ranges with low overall access of diagnostic test results include 12 to 17 years and older than 70 years. The institutional policy at UIHC limits proxy access to the patient portal for children aged 12 to 17 years; thus, efforts to increase portal usage in this age range have to be age appropriate. Another study has shown that older adults may be unaware of what medical information they can access via a patient portal, potentially limiting their enthusiasm for the use of the patient portal [55]. They may also be unaware of the different features available and the variety of ways that they can access information via patient portals. Training and assistance from family and friends may be helpful in overcoming resistance and lack of experience or comfort with the use of new technology such as patient portals [16,29,35,55].

Finally, this study shows that ED patient access of the diagnostic test results spans a wide time range, with some patients accessing results within minutes of availability in the patient portal (likely because of quick access to the patient portal following email or other notification that a diagnostic test result was available) and others only months after the ED encounter. Median view rates were between 2 and 4 days for laboratory and radiology results. There was a large spike that occurred in the 3 to 7 days range. This pattern is different from what we observed with viewing of results by outpatients at UIHC, where the most prominent spike in viewing is within 24 hours of availability in the patient portal, with a steady tail in viewing patterns after 24 hours [41,42]. For ED patients, there are a variety of factors that could influence viewing patterns. There could be delays from patients recovering from the cause of the visit before going and viewing results. If the patient is admitted to an inpatient unit from the ED, access to results would be influenced by factors such as internet access within the hospital and assistance from family and friends. Results from the testing performed in the ED could also become important later as patients have follow-up visits with outpatient providers. Finally, the nature of the patient’s illness likely has an impact on the use of a patient portal.

### Limitations

The limitations of this study include that data were obtained only from a single academic medical center ED with has a predominantly white patient population. Activation and access data for the patient portal also cannot capture the impact these data have on patient experience, especially as many patients seen at the institutional ED (which serves as the highest-complexity, level 1 trauma center in a wide geographic region) receive primary care at other health care institutions not affiliated with UIHC. In addition, as mentioned above, the data did not distinguish between patient or proxy access or the identity (eg, gender and age) of the person doing proxy access.

### Conclusions

We found that patient portal access of ED diagnostic test results was highest in the following populations: females, those with self-reported race as Caucasian or Asian, and those aged 0 to 11 years and 18 to 60 years. Groups with the lowest access rates included African American males, Hispanic/Latino individuals, teenagers, and those aged 81 years or older. Differences between groups in terms of diagnostic test access were less striking when looking at only those with activated patient portal accounts, suggesting that getting patients to activate a portal account is an important barrier. It is also important to note that even groups with the highest access rate viewed diagnostic test results at less than 20%, leaving much opportunity for improvement. More research should be done to ascertain the strategies for increasing patient portal usage in the ED. Data on patient portal usage can guide education effects to enhance patient engagement and minimize disparities between patient groups.

## Multimedia Appendix 1

Breakdown of data by self-reported race and age.

## Abbreviations

CT: computed tomography
ED: emergency department
EHR: electronic health record
HITECH: Health Information Technology for Economic and Clinical Health Act
MRI: magnetic resonance imaging
PCR: polymerase chain reaction
RWB: Reporting Workbench
UIHC: University of Iowa Hospitals and Clinics
