# Supplementary material for: The Use of an Electronic Health Record Patient Portal to Access Diagnostic Test Results by Emergency Patients at an Academic Medical Center: Retrospective Study
Source: J Med Internet Res. 2019 Jun 28;21(6):e13791. doi: 10.2196/13791 (PMC6625217; doi:10.2196/13791)
Supplement: Supplementary file 1 [file jmir_v21i6e13791_app1.pdf]

Supplementary Figures

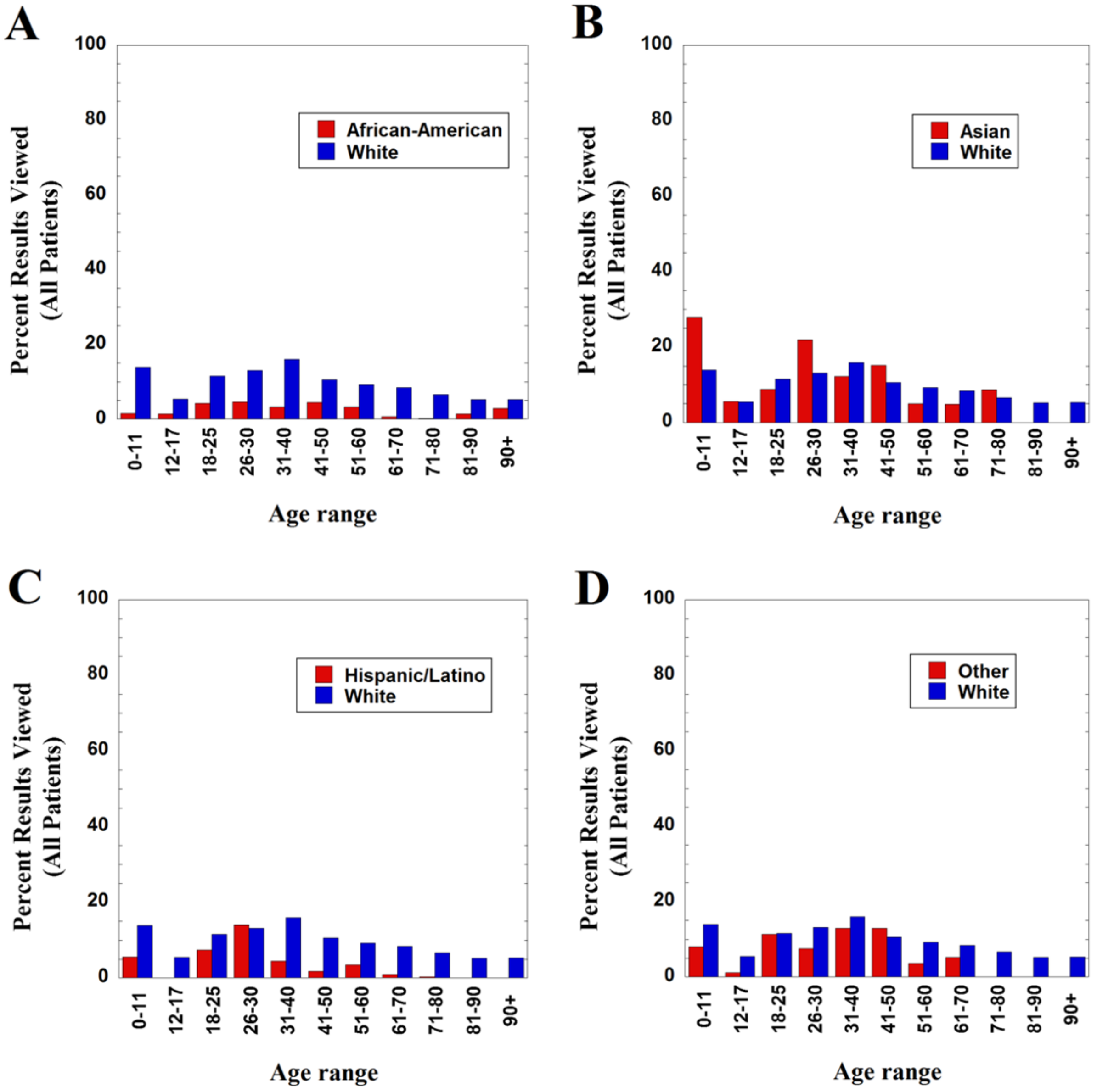

Supplementary Figure 1. View rates of emergency department laboratory tests and variation by age and self-declared race. Each panel compares a different self-declared race category (red bars) to White (blue bars). Population includes all patients who had at least one laboratory test performed during period of retrospective analysis. Data based on 25,361 unique patients (2,254 African-American/Black, 451 Asian, 1,257 Hispanic/Latino, 762 Other, and 20,637 White) and 208,635 tests. Bars show percent of results viewed within each sub-category.

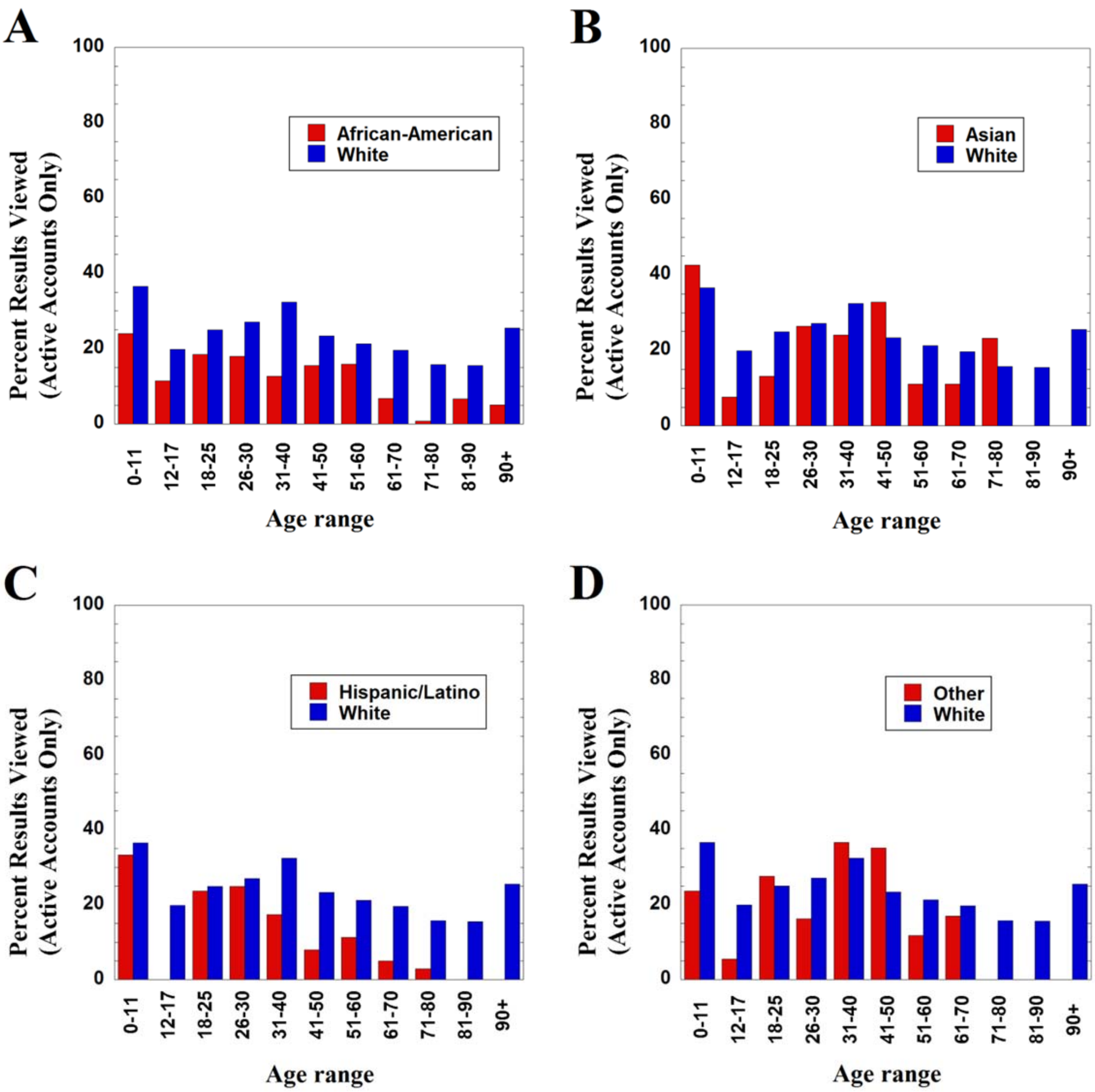

Supplementary Figure 2. View rates of emergency department laboratory tests and variation by age and self-declared race. Each panel compares a different self-declared race category (red bars) to White (blue bars). Population is limited to only those patients with active portal accounts and who had at least one laboratory test performed during period of retrospective analysis. Data based on 9,482 unique patients (491 African-American/Black, 262 Asian, 333 Hispanic/Latino, 241 Other, and 8,155 White). Bars show percent of results viewed within each sub-category.
